# Evaluating preclinical evidence for clinical translation in childhood brain tumours: Guidelines from the CONNECT, PNOC, and ITCC brain networks

**DOI:** 10.3389/fonc.2023.1167082

**Published:** 2023-04-05

**Authors:** Chris Jones, Karin Straathof, Maryam Fouladi, Darren Hargrave, Michael Prados, Adam Resnick, Francois Doz, David T.W. Jones, Sabine Mueller

**Affiliations:** ^1^ Division of Molecular Pathology, Institute of Cancer Research, London, United Kingdom; ^2^ Department of Oncology, University College London Cancer Institute, London, United Kingdom; ^3^ Developmental Biology and Cancer, University College Great Ormond Street Institute of Child Health, London, United Kingdom; ^4^ Pediatric Brain Tumor Program, Division of Hematology, Oncology, and Bone Marrow Transplant, Nationwide Children’s Hospital, Columbus, OH, United States; ^5^ College of Medicine, The Ohio State University, Columbus, OH, United States; ^6^ Haematology and Oncology, Great Ormond Street Hospital for Children, London, United Kingdom; ^7^ Department of Neurological Surgery, University of California, San Francisco, CA, United States; ^8^ Division of Neurosurgery, Center for Data-Driven Discovery in Biomedicine, Childrens Hospital of Philadelpia, Philadelphia, PA, United States; ^9^ SIREDO Centre (Care, Innovation and Research in Pediatric, Adolescent and Young Adults Oncology), Institut Curie and Univesity Paris Cité, Paris, France; ^10^ Hopp Children’s Cancer Center Heidelberg (KiTZ), Heidelberg, Germany; ^11^ Division of Pediatric Glioma Research, German Cancer Research Center (DKFZ), Heidelberg, Germany; ^12^ Department Neurology, Neurosurgery & Pediatrics, University of California, San Francisco, CA, United States; ^13^ Department of Pediatrics, University of Zurich, Zurich, Switzerland

**Keywords:** pediatric, CNS, preclinical, models, translational, *in vitro*, *in vivo*

## Abstract

Clinical outcomes for many childhood brain tumours remain poor, despite our increasing understanding of the underlying disease biology. Advances in molecular diagnostics have refined our ability to classify tumour types and subtypes, and efforts are underway across multiple international paediatric neuro-oncology consortia to take novel biological insights in the worst prognosis entities into innovative clinical trials. Whilst for the first time we are designing such studies on the basis of disease-specific biological data, the levels of preclincial evidence in appropriate model systems on which these trials are initiated is still widely variable. We have considered these issues between CONNECT, PNOC and ITCC-Brain, and developed a framework in which we can assess novel concepts being brought forward for possible clinical translation. Whilst not intended to be proscriptive for every possible circumstance, these criteria provide a basis for self-assessment of evidence by laboratory scientists, and a platform for discussion and rational decision-making prior to moving forward clinically.

## Introduction

Despite remarkable advances being made in the treatment of numerous paediatric cancers over the past 40 years (1), tumours of the central nervous system remain the biggest cause of cancer-related death in children and young adults (2). For many entities, survival rates have remained unchanged for decades, and represent a major unmet clinical need (3, 4). Previous generations of clinical trials were necessarily based on an incomplete appreciation of the unique biology of childhood brain tumour entities, and a lack of preclinical evidence in appropriate model systems to show that they were likely to be effective. The failure of these studies, therefore, can now been seen as not unexpected (5). In recognition of this devastating societal impact, over the past 10-15 years partnerships between patients/families, clinical, translational and laboratory-based scientists worldwide have dramatically improved access to tissue and funding for childhood brain tumour research, which coupled with the rapid advances in next-generation sequencing and other molecular profiling techniques, has revolutionized our understanding of the underlying biology of a plethora of childhood brain tumours (6–9).

In the recent 2021 5^th^ Edition of the WHO CNS Classification of CNS tumours, a large proportion of entities were recognized as being of paediatric ‘type’, or occurring largely in the children and young adult populations (10). Underscoring this delineation is the integrated diagnostic approach which includes key distinguishing biological data, and the appreciation of distinct drivers of the childhood disease types and the subtypes within (11). For many of these tumours, we now have both novel targets for therapeutic development, and a framework by which these children may be stratified for clinical trial enrollment, which will lead to better response assessment in a molecularly defined context. There is still substantial uncertainty, however, around the amount and type of preclinical data that is needed to develop trials that are more likely to succeed compared to their predecessors. The key tenet for moving a concept into the clinic is a strong biological rationale, with support from robust preclinical data in appropriate model systems (12). Until recently, these have been difficult to achieve, which coupled with unselected patient populations has likely contributed to the lack of success of clinical trials for children with brain tumours. As several international paediatric neuro-oncology clinical trials consortia have emerged to address the clinical issues, we now need international consensus in developing more robust preclinical platforms to provide data packages that can be reviewed objectively and systematically prior to clinical implementation.

## International childhood brain tumour consortia

The present article is a result of discussions between three international paediatric neuro-oncology clinical trials consortia. Each has a slightly different focus, approach, tumour-type or discipline expertise, and geographical footprint. The groups work non-competitively to ensure access to the most promising trials in the most appropriate environments, and have a degree of overlap in key personnel and centres worldwide.

### CONNECT

CONNECT (the COllaborative Network for Neuro-oncology Clinical Trials) is a collaborative of 18 international sites across North America, Europe, UK, and Australia, with expertise in paediatric brain tumour research and clinical trials. Its purpose is to conduct scientifically rational pilot studies to assess feasibility and early efficacy of incorporating promising novel agents to established frontline therapeutic regimens in children with newly-diagnosed, high-risk brain tumours. CONNECT serves as a clinical research organisation providing concept and protocol development, data and study management, drug shipping, and all operational support. It has a diverse portfolio of trials in different childhood brain tumour entities, partnering with multiple drug companies and foundational supporters. The clinical network is supported by an active Preclinical Group, whose goal is to provide scientific assessment of novel concepts brought to the consortium for clinical translation, and to assemble collaborative research teams to provide additional experimental data as warranted.

### PNOC

PNOC (Pacific Pediatric Neuro-Oncology Consortium) is an international clinical trial consortium with 22 sites in the US as well as sites in Switzerland, Israel, Netherlands, Canada and Australia with recent expansion into Germany, Egypt and India. The mission of PNOC is to develop biology driven trials and expand access to innovative therapies globally for children and young adults with brain tumors, Development of clinical trials is supported by disease specific working groups composed of clinical, translational, imaging and basic science experts spanning key entities such as high grade glioma/diffuse midline glioma; ependymoma, germinoma, medulloblastoma and craniopharyngioma amongst others. Data collected as part of PNOC trials – such as imaging and genomic data – is shared in real-time with the research community through collaboration with the Children’s Brain Tumor Network (CBTN).

### ITCC Brain

ITCC Brain is the CNS tumor-specific working group of ITCC (Innovative Therapies for Children with Cancer), a consortium of over 60 expert pediatric oncology centers and 25 leading research laboratories from across Europe. ITCC Brain aims to provide a framework for bringing together biologists and clinician scientists generating cutting-edge basic and translational research findings, with clinicians in large early-phase clinical trial centers, in order to accelerate the translation of novel science into effective new treatments for children with brain tumours. ITCC Brain has a portfolio of investigator-initiated trials as well as providing support for industry-led studies, and is working to expand this portfolio through well-planned studies based on strong preclinical data. The group also works closely within the larger ITCC organization to participate in entity-agnostic, biomarker-driven studies; and the group also benefits from other ITCC-led initaitives such as the ITCC-P4 pre-clinical platform [ref] and an upcoming platform for integrating data across international pediatric precision oncology progams.

## Guidelines for new concepts

Prior to the initiation of a clinical trial, we believe a robust process should be in place to critically review extant preclinical data which support the concept, as well as the strategy for clinical implementation. The intention here is not to produce binary ‘go/no-go’ decisions, but rather to assess whether a threshold of evidence has already been passed for which the clinical need mandates the concept moving forward. This may necessarily be different for distinct target patient populations, and if certain data are felt to be lacking, constructive and realistic suggestions should be made as to how to build confidence in the approach. Independent reviewers will be asked to judge any new concept proposal based on clinical significance, trial design including embedded correlative studies (*e.g.* CNS penetration; molecular profiling; subtype responses *etc.*) as well as feasibility in the context of competing trials and available patient population to conduct the proposed study. A strong biological rationale is required, with preclinical evidence benchmarked against specific idealized guidelines, with justification for any criteria not explicitly met. In this perspective, we will focus mostly on the preclinical aspects, but stress that these data dovetail with a careful, and early inclusion of the following clinical considerations:

### Clinical significance

The concept should address a clear unmet need, and represent a novel therapeutic development. This could be in the upfront setting, when an effective standard of care (SOC) has not been developed, for tumours with an extremely short overall survival, such as paediatric-type diffuse high-grade glioma (PDHGG), in particular diffuse midline glioma (DMG); subtypes of medulloblastoma (especially Group 3/4 or SHH, TP53-altered), atypical teratoid/rhabdoid tumours (ATRT), embryonal tumours with multilayered rosettes (ETMR) and others. This could also include tumours for whom the current SOC is associated with long-term burden in terms of quality of life (QOL), such as craniopharyngioma, ependymoma *etc.*

### Trial design

The concept should clearly outline the primary, secondary and exploratory aims as well as endpoints. There ought to be a valid statistical design, with innovative models to be encouraged to maximise the information gained from a minimal number of patients. It should be clearly stated how correlative studies will be used to interpret successes and failures, with inclusion of plans for access to tumour tissue (including type of material, and regulation of storage and availability for follow-up studies), genomic profiling (either as part of the study or per SOC), and digitized histology and radiology (with access and governance details). Plans for CSF and plasma/serum collection (if feasible), and details of functional (cognitive outcomes; vision, endocrine, QOL dependent on disease subtypes) and imaging endpoints ought to be provided. There should be a strategy for obtaining post-treatment tumour tissue including autopsy collection protocols, and plans for appropriate analysis of such tissue. In addition, over the last few years efforts have been made to harmonize clinical trial endpoints across consortia which will allow for more direct comparison between different study therapies. Harmonizing correlative study endpoints and biological correlates will further inform cross trial comparison within a specific disease context.

### Data sharing

Data should be made accessible in real time to the research community without compromising clinical trial endpoints. This is of critical importance across our consortia (and others) to ensure rapid dissemination of both positive and negative data, application of important lessons learned, and provide a means for cross-validation of results, improving ongoing trial design, and identifying appropriate patient populations for trial inclusion beyond traditional research silos.

### Feasibility

Documentation should be provided where other compounds in the same mechanism of action class have already been evaluated clinically (or preclinically) for the given or related indications. There ought to be a plan for access of the relevant patient population within the footprint of the clinical trials consortium to which the application is submitted; there should also be an evaluation of other competing trials within the consortium’s portfolio, and that of other consortia.

## Principles of preclinical assessment

We strongly recognize that there is no ‘one size fits all’ approach to the process of assessment, and that each concept should be judged on its own merits on the basis of the specific clinical need of the patient populations proposed, and the feasibility of generating the idealized preclinical data package in such a context. We therefore indicate signposts for what a strong concept proposal should ideally include, and have identified five principles that could guide assessment of a data package presented for consideration (**Table 1**).

**Table 1:** *Principles of preclinical assessment*.

Clearly defined target population(s) based upon mechanism of action
Efficacy in multiple relevant models *in vitro* and *in vivo*
Safety assessment of off-tumour target expression, particularly for immunotherapies
Data showing penetration into tumour tissue at clinically relevant doses
Demonstration of on-target effect at clinically achievable doses and availability of predictive biomarkers

Guidelines for consideration of a preclinical data package for clinical translation.

Firstly, there should be a clearly defined target population identified, based upon the mechanism of action of the investigational agent(s). In some instances, this will be relatively wide, and may span tumour entities and genotypes, whilst in others a highly restricted set of patients may be targeted. In terms of preclinical assessment, distinction is not made on this basis, so long as convincing rationale is provided. Secondly, evidence of efficacy of the agent in multiple relevant preclinical models, both *in vitro* and *in vivo*, is sought. The term ‘relevant’ here is to be contexualised by the investigators and reviewers, and may depend on disease biology and/or the agent being tested. There is complete recognition that multiple models may not be available for all tumour types or subgroups; here explanation and justification need simply be provided for both the number and identity of models chosen. Thirdly, there should be an assessment for the therapeutic window and potential safety issues by reporting target expression in the non-tumour compartments, through analysis of either novel or published data. This is of particular relevance for agents targeting wild-type targets, and for immunotherapies. Fourthly, data should be provided showing penetration of the agent into tumour tissue at clinically relevant doses; again this could be newly-generated data by the investigators or from the literature/drug company internal data, with recognition that extrapolation of doses from *in vitro* assays is imperfect (13). Finally, there should be demonstration of an on-target effect of the agent at clinically achievable doses in a relevant model system. Assays developed to assess this should also be evaluated for their ability to serve as predictive biomarkers for trial inclusion and/or post-hoc response assessment.

**Figure 1:**
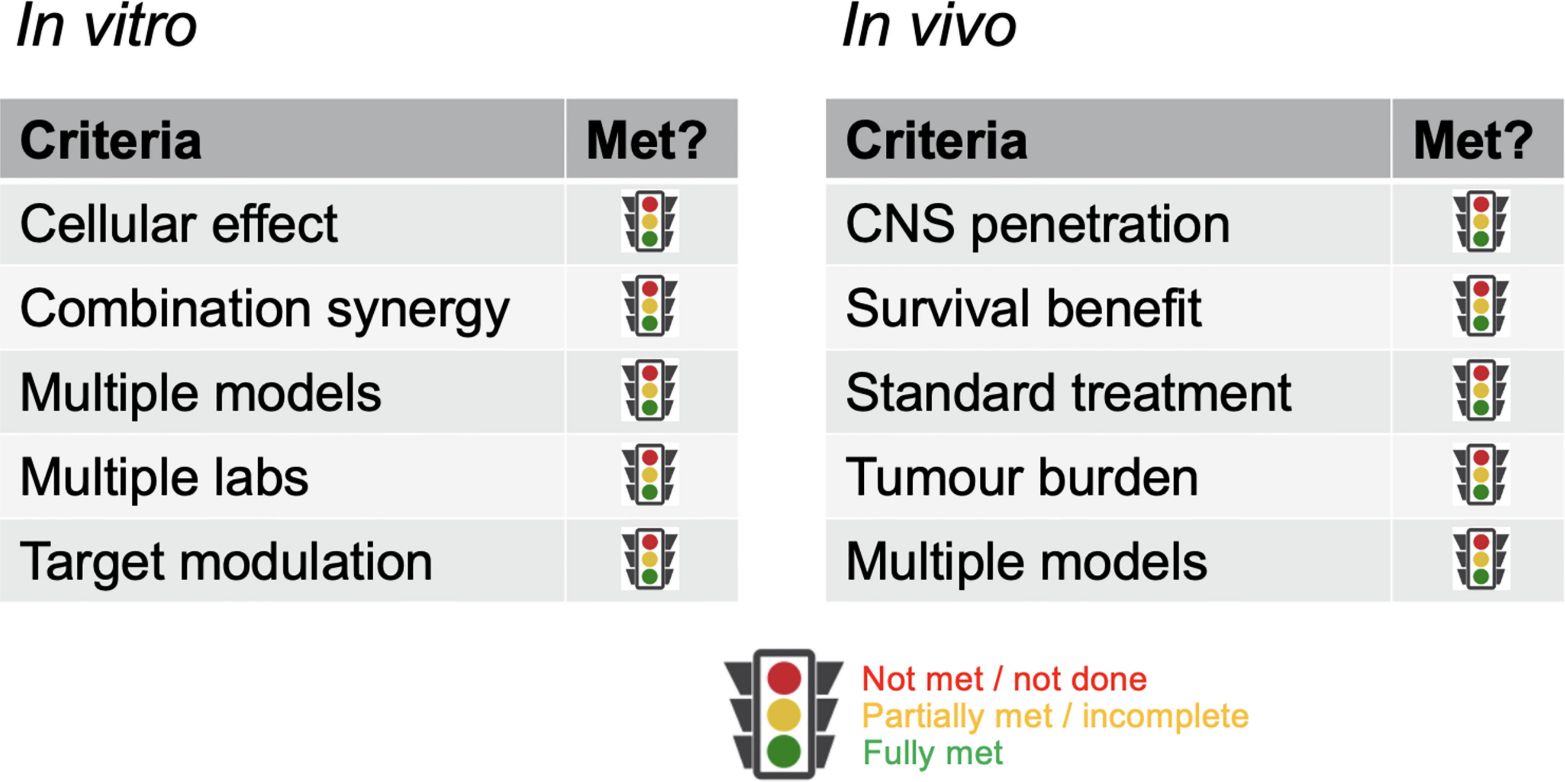
*Criteria for critical review.* Key parameters against which data packages should be assessed. Rather than a metrics-based scoring system, a fully justified benchmarking against each category should be provided.

With such overarching principles driving the initial consideration of the suitability of a new concept being ‘ready’ for clinical translation, we provide specific assessment criteria in respect of *in vitro* and *in vivo* evidence that would aid prioritization of ideas. As previously stated, it is recognized not all circumstances will allow for all criteria to be met; where they cannot, the guidelines are meant to serve as discussion points rather than reasons for exclusion (**Figure 1**).

### Specific *in vitro* criteria

◼ The agent to be tested should be potent in the models tested, with evidence of a clear cellular effect in terms of cell viability, cell death, cell differentiation or other appropriate end-points. This may be demonstrated in terms of effects observed (IC_50_/GI_50_ *etc.*) at sub-micromolar concentrations and/or showing a greater than two-fold statistically significant differential sensitivity in models representing the target population compared to (i) an appropriate ‘normal’ cell type and/or (ii) other disease subtypes and/or (iii) other disease entities.
◼ Assays should be carried out in multiple appropriately-accredited models representing the heterogeneity of the target population(s). Where available for a given entity or subtype *etc.*, this should be carried out in at least n=4-6 distinct models, with phenotypic/genotypic data for each provided. Where available, both patient-derived and genetically-engineered models are desirable for a given target.
◼ There should be evidence of target modulation at doses producing a cellular effect by an appropriate assay, western blot, ELISA, mass spectrometry, *etc.*) in at least n=2 models. Although not a prerequisite for preclinical assessment, indications should be given as to the applicability of such an assay that is translatable to the clinical setting a predictive biomarker.
◼ For immunotherapies, evidence should be provided of target antigen-specific tumour cell lysis, including where possible of (primary) tumour cells with endogenous target antigen expression. Here, level of target expression reflective of that of primary tumours should be taken into account given that immunotherapeutics commonly have a target density threshold for efficacy (14).
◼ For combination studies, there should be evidence of at least additivity, or better formal synergy, of the agents to be combined by one or more appropriately designed assays, including but not restricted to the Chou-Talalay median effects model (15), BLISS independence score (16), isobologram (17) *etc.*). This should be carried out in at least n=2 models, where possible.
◼ Collaborative studies across laboratories are encouraged in order to demonstrate reproducibility, as well as maximise resources and expand the number of models available. Where data is pooled in such a way, at least n=1 of the models/assays should be consistently assessed across all partner laboratories in order to assess comparability.

### Specific *in vivo* criteria

◼ If clinical data from human studies is unavailable, demonstration of drug penetration into the relevant normal brain and/or tumour tissue of appropriate model organisms. These experiments should be carried out at tolerable doses resulting in concentrations at least greater than the *in vitro* IC_50_/GI_50_ values, assessed by direct measurement (using assays such as LC-MS, MALDI-TOF, *etc.*) and/or appropriate biomarker modulation (*e.g.* western blot, immunohistochemistry, *etc.*).
◼ For immunotherapies, if clinical data from human studies is unavailable, demonstration of homing to and penetration into tumour tissue of appropriate model organisms at tolerable doses should be provided. Presence of the immunotherapeutic agent at tumour sites can be demonstrated by immunostaining using e.g. anti-idiotype antibodies or detection of linked marker genes).
◼ There should be demonstration of a statistically significant survival benefit of treated animals, typically >20% prolongation of the median survival over vehicle (or control biologic)-treated controls. For combination studies, in addition, a statistically significant survival benefit for the combination of >10% of the median survival over the most effective single agent should be seen.
◼ As for *in vitro*, assays should be carried out in multiple appropriately-accredited where available. This should be undertaken at least n=3 distinct models representing the target population(s), and grown in the relevant orthotopic location where feasible. Where available, both patient-derived and genetically-engineered models, with at least n=1 in an immunocompetent background, are desirable. These could be carried out in models in the same or different species, with the latter encouraged.
◼ There should be evidence of a statistically significant reduction in tumour burden on treatment provided, assessed by an appropriate assay (*e.g.* MRI, bioluminescent imaging, ddPCR *etc.*). As with *in vitro*, indications should be given as to clinical translation of any predictive biomarkers.
◼ Priority will be given to treatments which can be shown to provide a survival advantage greater than SOC treatments for a given patient population, in a preclinical trial mimicking the appropriate clinical protocols (18). This could include addition and/or comparison to a standard radio/chemotherapy regimen, including surgical resection where practicable, as well as ‘mouse hospital’ designs of multiple individual patient-derived models at, *e.g.* n=1 mouse each (19).

## Application

Within our consortia, elements of these principles have been generically applied since initiation, but not in a systematic way. By formalizing standards, we aim to achieve two things. The first is to provide an unbiased methodology for assessing concepts brought forward from multiple sources and across disparate entities and therapeutic targets, such that cross-review between the various collaborative groups can be undertaken to the same criteria. Such a harmonized but flexible approach also seeks to provide investigators with a clear set of guidelines against which they may judge their own extant data, and help to plan additional experimental work. Inherent in this is a desire to encourage and facilitate data sharing to avoid unnecessary duplication of effort. The second critical goal is to provide a framework for discussion of novel concepts, rather than a strict metrics-based exercise. A key point is that that concepts may come from many different sources, and certainly external to any of our consortia (or others). It is the hope that having such guidelines would encourage researchers not otherwise connected to such groupings to self-evalaute their own data prior to engagement with clinical trials groups, but not in any exclusionary way; the hope is to stimulate discussion and not to restrict good ideas being brought forward at any stage.

We recognize the present limitations inherent in certain fields which make adherence to certain points impossible, and aim to highlight these caveats for frank conversation as to their importance relative to the other evidence presented, and clinical need of the target population. In this way, we also hope to flag areas that are in need of further development by the field. It should also, however, hold to account other areas in which the criteria could be, but are often not, routinely met. An example is in the desire for demonstration of efficacy and on-target effects *etc.* in multiple disease models. We appreciate that for certain high-risk childhood brain tumour entities such as ETMR and ATRT they may be limited (20, 21), or for others like Group 3/4 medulloblastoma they may be imperfect representations of the human disease (22). For others such as DMG however, large panels of patient-derived cells, PDXs and GEMMs are widely available, and single cell line studies are hard to justify (23–25). In all cases, we anticipate an iterative process whereby the criteria provide a checklist for early discussion, template for initial benchmarking, and a guidebook for eventual translational decision-making.

## Limitations and challenges

The proposed criteria are intended as positive and achievable, with the goal of leading to therapies that are more likely to be successful in the clinic (26, 27). They are meant to encompass the most common treatment concepts brought forward to our consortia, and will likely need refinement to include more innovative modalities. We do not expressly provide specific proposals for how to assess novel drug delivery methodologies, for example, including such disparate approaches as nanoparticles (28), convection-enhanced delivery (29) and focused ultrasound-mediated opening of the blood-brain barrier (30), *etc.* Another emerging area which may require distinct end-points clinically, and therefore unique criteria preclinically, is that of cancer neuroscience (31). We do not explicitly lay out a framework for assessing the modulation of cancer cell – neuron interactions, nor what our expectations should be in the preclinical context for such agents to have a beneficial effect in patients. The same could be said to be true of other microenvironment modulation strategies, such as targeting tumour-associated immune cells or angiogenesis (32). Here, novel model systems such as *ex vivo* tumour explant or organotypic models which recapitulate the complex tumour milieu are being developed to provide information complementary to that of current models (33, 34). Further refinements to our *in vivo* strategy will likely come to include a more thorough consideration of the age of animals used for such studies, to better replicated the developmental context in which these tumours arise (35), as well as evaluating therapies in both male and female models, given the sex-related biological differences which are beginning to emerge (36). Moreover, as we aim to develop therapies which can spare children and young adults from the toxic effects of chemotherapy and radiation, we would need to include additional means to assess how we improve QoL measures and control for the late effects of therapies (27).

As our biological understanding of paediatric CNS tumours increases, and we subclassify them into ever-more subtypes with distinct drivers warranting unique therapies (37), we face a challenge both in terms of generating preclinical data and moving these concepts into clinical trial. Idealised criteria in which we hope to see evidence of efficacy in multiple models, *in vitro* and *in vivo*, makes little sense for ultra-rare, newly-defined subtypes, and patient numbers for such entities mean traditional clinical trial designs are unlikely to recruit sufficient numbers, even with international co-operation. It will be a challenge for the community to determine to what extent we can relax or refine our standards to assess novel concepts in these entities, and how they may be robustly and safely tested in patients. We need also to be cogniscent that positive results in model systems do not necessarily predict for success in clinical trial (38–40). Although we assume that the patient-centric and biologically-driven models we have recently developed will be a substantial advance on what went before, this is as yet unproven. Careful credentialling of the models (and assays) required as part of the criteria are inherent in generating preclinical data which will eventually prove effective in the clinic, and need constant assessment and benchmarking in the manner of the criteria we apply here to the data generated with them.

## Data availability statement

The original contributions presented in the study are included in the article/supplementary material. Further inquiries can be directed to the corresponding author.

## Author contributions

CJ, KS and SM jointly conceived, wrote and edited the article; all other authors wrote and edited the article. All authors contributed to the article and approved the submitted version.
